# Differential Usage of Learning Management Systems in Chemistry Courses in the Time after COVID-19

**DOI:** 10.1021/acs.jchemed.2c00850

**Published:** 2023-04-21

**Authors:** Ying Guo, Daniel Lee

**Affiliations:** † Department of Chemistry, School of Science and Technology, Georgia Gwinnett College, 1000 University Center Lane, Lawrenceville, Georgia 30043, United States; ‡ STEM Academy, George Walton Comprehensive High School, 1590 Bill Murdock Road, Marietta, Georgia 30062, United States

**Keywords:** Administrative Issues, First-Year Undergraduate/General, COVID-19, Learning management systems, LMS, Second-Year Undergraduate, Upper-Division Undergraduate

## Abstract

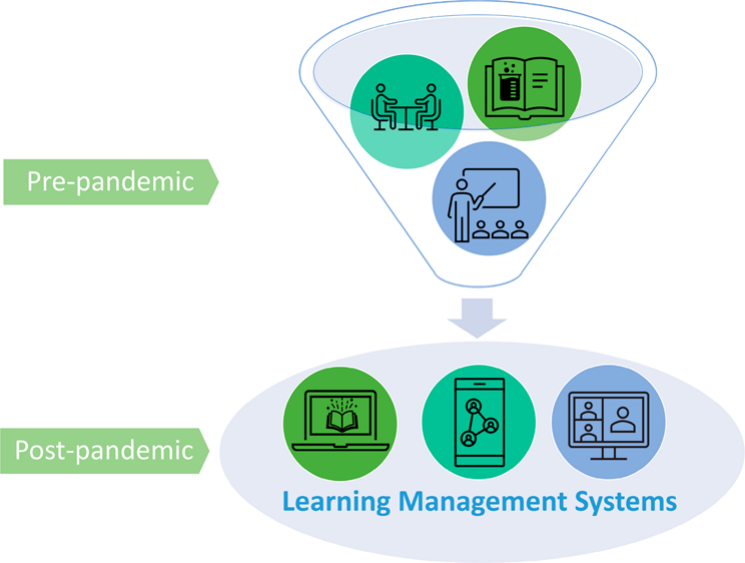

Learning management systems play a crucial role in addressing pedagogical challenges imposed by the COVID-19 pandemic. The solutions provided by the learning management systems (LMS) facilitated online instructions and helped form a community of learning and support. With the rapid increased usage during the pandemic and the return to face-to-face post-pandemic, an in-depth analysis on lasting changes in students’ engagement and the instructors’ use of the systems during and after the pandemic is needed. This study aims at providing the analysis results on the differential usage of the learning management systems in a chronological time frame and on a course-level-specific aspect. Analysis conducted on the LMS usage data of chemistry courses between Fall 2019 and Fall 2021 suggests unique patterns, depending on the course levels. The extent of students’ interaction with peers and course materials varied for different course levels. The degree of usage of learning management systems by instructors also depended on the course levels. Instructors in lower-level courses (1000 and 2000 level courses) continued to use learning management systems extensively after the pandemic, while instructors in upper-level courses (3000 and 4000 level courses) rebounded to their pre-pandemic level of usage after resuming face-to-face instructions.

## Introduction

Learning management system (LMS) is software to deliver, track, and manage training and/or education. The LMS includes a wide range of systems from one for managing educational records to that for delivering and distributing courses over the Internet.^1^ Such systems are managed by the administration of educational institutions and facilitate access to the learning content.^2^ As pedagogical challenges are addressed through the technological solutions that LMSs can provide, the LMSs accessible via the Internet reflect a paradigm shift and have been adopted rapidly in manly educational institutions. With the outbreak of COVID-19, the demand in the LMS rapidly increased globally,^3^ and the global e-learning market share is expected to exceed $370 billion in 2026.^4^

Georgia Gwinnett College (GGC) is an open-access public liberal arts college located in Lawrenceville, Gwinnett County, Georgia. GGC was founded in 2006 as the first four-year college founded in Georgia in more than 100 years and the first four-year public college created in the U.S. in the 21st century.^5^ Gwinnett County is the second most populous county in the state and is the seventh most ethnically diverse county in the state according to 2021 census data. GGC’s student body reflects a similar ethnic diversity, classified as a Hispanic Serving Institution with 25% Hispanic/Latino population,^6,7^ and the institution has been a minority majority institution, as demonstrated by the corresponding demographic information.^8^ GGC combines student–faculty engagement practices with small class sizes, individual attention, a diverse and inclusive culture, and student mentoring to enhance student success.^5^ Most of chemistry courses including lecture–lab combined ones (so-called K courses) adopt a single-instructor model, in which a single set of classmates and a single instructor form a community of learning and support.

Chemistry courses were delivered in person prior to the COVID-19 pandemic. For the K courses, the lecture and lab constituted a section, and the section was run by a single instructor without a teaching assistant. The size of enrollment was capped at 24 students, and the interaction between students and the instructor was maintained as interactive and significant throughout a semester. With the outbreak of the COVID-19 pandemic, the modality of instructions was diversified either to a completely online delivery or a hybrid format. The online courses were further varied into synchronous and asynchronous. Later, a certain number of in-person courses or sections were revived. Therefore, there would be four types of course delivery (in-person, hybrid, online synchronous, and online asynchronous) in a semester.^9,10^ During the pandemic, there were times when the enrollment for a section was temporarily increased to 28 students, which returned to 24 afterward.

Online delivery inevitably called for changes in specific methods of dissemination of lecture and laboratories.^11,12^ Virtual instructions necessitated recording lecture and a portion of laboratory to provide students with lectures and visualized information on laboratories. For asynchronous sections, it was the only method adopted for the course. Consequently, relevant adaptations became crucial in assessments and lab curricula in many courses.^13,14^

In Fall 2021, the GGC campus resumed normal onsite operations where most courses returned to in-person delivery modality with limited number of sections offered as hybrid or online (synchronous or asynchronous). As of Fall 2022 semester, among all 89 sections offered in the chemistry department, 20 sections are taught in these modes: nine hybrid, eight online synchronous, and three online asynchronous sections. All of these courses are 1000 level courses (CHEM 1151K, CHEM 1152K, CHEM 1211K, and CHEM 1212K), while the upper-level courses are in a face-to-face mode. The detailed list of courses can be found in the Supporting Information Table S1.

Prior to the pandemic, Brightspace (Desire to Learn; D2L), the LMS employed at GGC, was used primarily for simple purposes such as course communication, assignment collection, and gradebook. D2L uses Blackboard Collaborate for online “virtual classroom” video conferencing. There were phases of varied extent of LMS usage as the transition from completely face-to-face instructions to partially or fully online modes. The partial transition took place in March 2020,^15^ and the Summer 2020 semester adopted fully online modality over all chemistry courses. With the onset of the COVID-19 pandemic, the LMS began to play more essential functions.^16,17^ The whiteboard and the Breakout Groups features were utilized in delivering real-time lectures and group discussion and problem solving to mimic a typical in-person classroom environment. Assessments including quizzes and exams were administered through the LMS. Recordings of lectures with transcripts were provided on D2L for students to watch either for review or for catch-up at a later time as well. The integrity of the academic evaluation of students’ performance was supported by a custom interface for web browsers during the assessments. Respondus LockDown Browser and/or Monitor were adopted for GGC’s LMS as a platform compatible with the Brightspace.^9^

As it has been more than two years since the pandemic started and the instructions return to face-to-face in the post-pandemic time, it would be meaningful to investigate whether the pedagogical changes and adaptations made at the beginning of and during the pandemic persist after the pandemic, in particular, in terms of the utilization of LMSs. Since faculty and student acceptance toward LMSs varies,^18^ two specific research questions were proposed to delve in:

Research question 1: How does the level of student engagement with course materials and peer students change on LMS before, during, and after the pandemic?

Research question 2: How does the level of instructor utilization of LMS change before, during, and after the pandemic?

Answering these questions will provide the instructors and the broad chemical education community with relevant perspectives on the course-level specific course design and the LMS usage.

## Data Collection

An Institutional Review Board (IRB) was submitted to access archival data sets of all students who took chemistry courses between the Fall 2019 semester and Fall 2021 semester at GGC (located in USA). After the IRB was approved, a data request was submitted to the campus LMS administrator. A total of 8,751 entries of LMS usage data were returned with the following features: *Year, Semester, Course Code, Number Of Logins To The System, Content Completed, Content Required, Checklist Completed, Total Time Spent In Content, Quiz Completed, Total Quiz Attempts, Discussion Post Created, Discussion Post Replies*, *Discussion Post Read, Last Discussion Post Date*, and *Number Of Assignment Submissions*. The LMS usage data were normalized with the min–max normalization method.^19^ To be specific, each feature was normalized using its maximum and minimum values (see the equation below). The normalized data were then aggregated by semester and course level. The average of the normalized data was then reported and used in the subsequent analyses. The normalization was intended to scale the data to a known range and can improve the accuracy of data analysis for comparison.^20−22^ All analyses were completed using Jupyter Notebook 6.0.3.
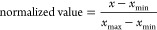


## Enrollment of Students in Chemistry Courses

A total of 20 courses were grouped into three categories based on the audience of courses: (1) 1000 level courses that are required for all students in the School of Science and Technology/School of Health Sciences, (2) 2000 level courses that are designed for chemistry and several closely related majors and minors such as biochemistry, and (3) 3000 and 4000 level courses that are primarily offered for chemistry majors. All 3000 and 4000 level courses were grouped together considering the small number of sections offered each semester compared to that of 1000 and 2000 level courses. A detailed list of courses in each category can be found in Supporting Information Table S1.

The numbers of students enrolled in different levels of chemistry courses from the Fall 2019 to Fall 2021 semesters are summarized in Table 1. The total enrollment of students in all chemistry courses decreased from 2082 in Fall 2019 to 1521 in Spring 2021 semester. In Fall 2021, the enrollment bounced back slightly up to 1572. Summer semesters were excluded from the study as the enrollment data included high percentage of transient students who were not enrolled at GGC during spring and fall semesters.

**Table 1:** Enrollment of Students in Chemistry Courses from Fall 2019 to Fall 2021

year	semester	total	1000 level courses	2000 level courses	3000 and 4000 level courses
2019	Fall	2082	1720	230	132
2020	Spring	1785	1458	216	111
2020	Fall	1791	1440	222	129
2021	Spring	1521	1244	188	89
2021	Fall	1572	1272	185	115

**Figure 1:**
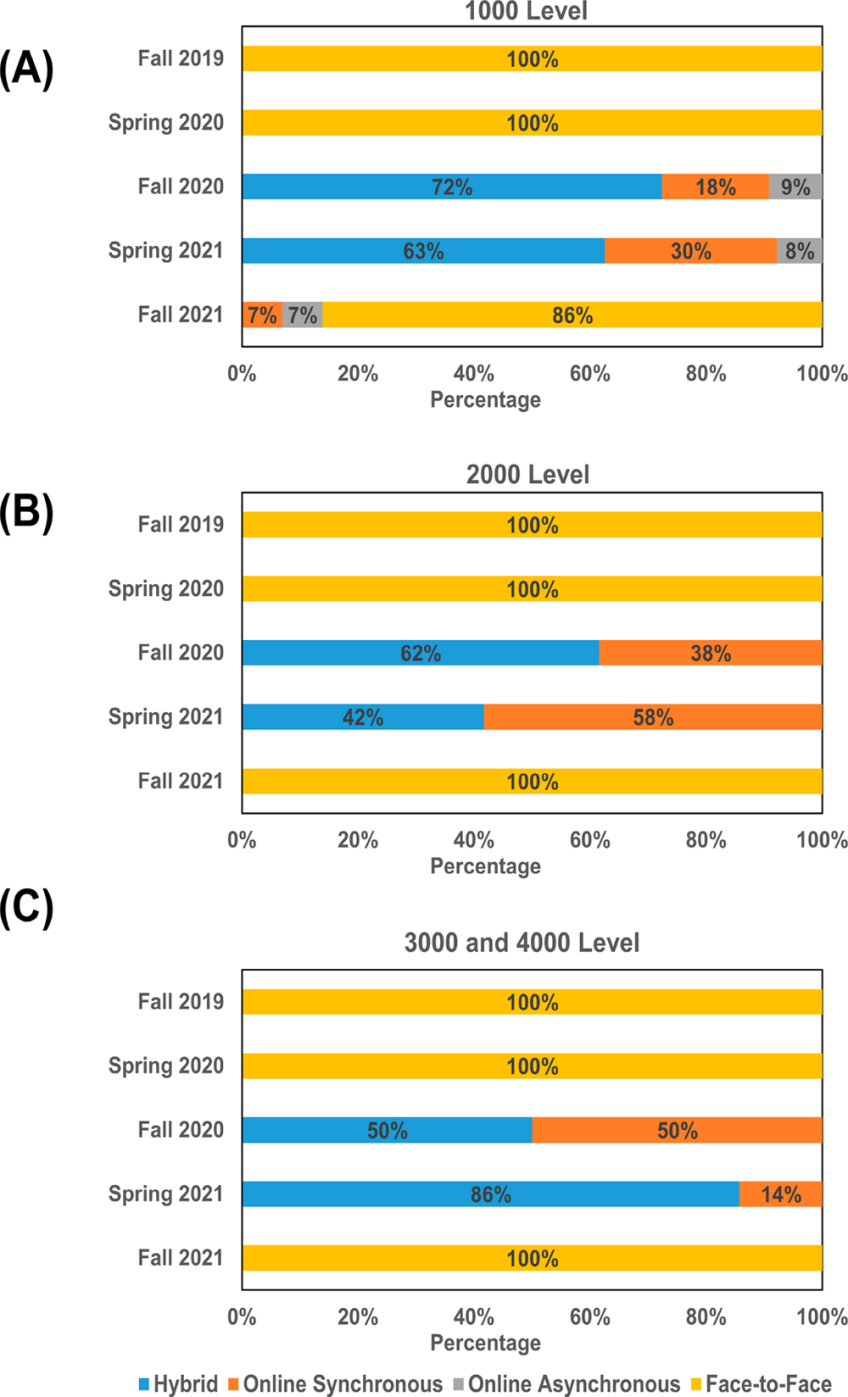
Number of chemistry courses offered in different modalities from Fall 2019 to Fall 2021 semesters for (A) 1000 level courses, (B) 2000 level courses, and (C) 3000 and 4000 level courses. Note that all courses in Spring 2020 transitioned to online synchronous and asynchronous in March 2020.

A further breakdown of sections offered in different modalities (face-to-face, hybrid, online synchronous, and online asynchronous) for each level of the courses is presented in Figure 1. In Fall 2019 before the pandemic, all courses were in a face-to-face mode. In Spring 2020, all sections started from the face-to-face modality but transitioned to online synchronous/asynchronous in March 2020 in response to the COVID-19 pandemic outbreak.^15^ In Fall 2020 and Spring 2021 semesters, a mix of hybrid and online sections was offered. For 1000 and 2000 level courses, a higher percentage of sections was offered online in Fall 2020 than in Spring 2021. However, the percentage of online sections in the 3000 and 4000 level courses dropped from 50% in Fall 2020 to 14% in Spring 2021. In Fall 2021 when GGC returned to normal operations, only 14% of 1000 level courses were delivered online, and all of the 2000, 3000, and 4000 level courses returned to entirely the face-to-face modality.

## Usage of LMS in All Chemistry Courses

**Figure 2:**
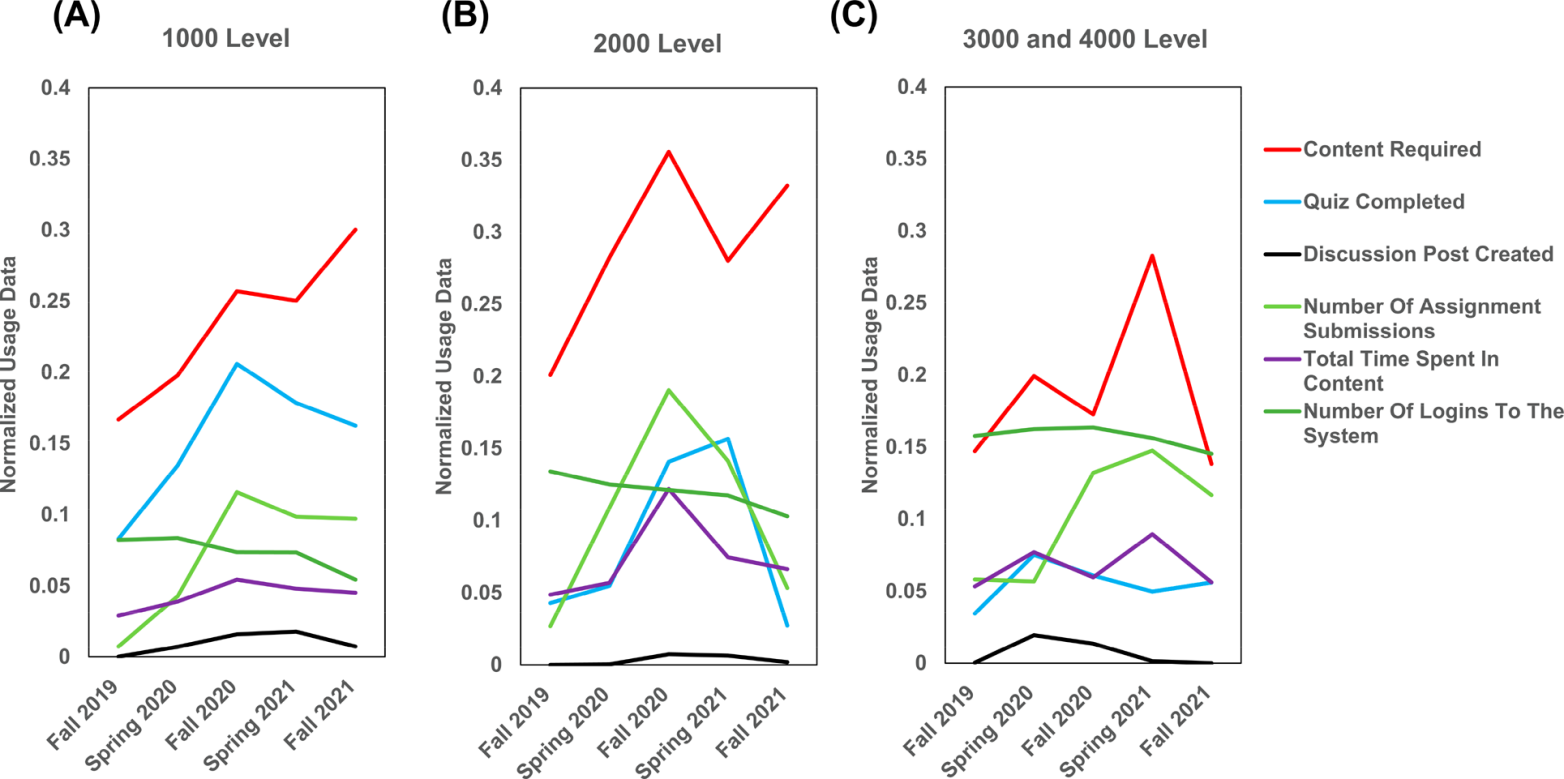
Normalized average usage of LMS features for (A) 1000 level courses, (B) 2000 level courses, and (C) 3000 and 4000 level courses from Fall 2019 to Fall 2021 semesters.

Six features were selected among all available features to represent the engagement of students with materials covered (*Quiz Completed*, *Number of Assignment Submissions*, *Total Time Spent in Content*, and *Number of Logins To The System*), students’ interaction with peer students (*Discussion Post Created*), and instructors’ utilization of LMS (*Content Required*). Detailed description of features can be found in the Supporting Information. The usage for each selected feature was normalized using the min-max rescaling method. After normalization, the respective usages of the LMS features are plotted in Figure 2. Detailed information about all features can be found in Supporting Information Tables S2–S4. These average normalized values represented the average usage level of different LMS features, and served as proxies to measure the level of student engagement and the level of instructor utilization of the LMS.

*Quiz Completed* and *Total Time Spent in Content* displayed similar trends. The courses at the 1000 level had the highest number of quizzes completed during the study period. In 1000 level courses, the number of quizzes completed by students and the total amount of time spent in content both increased during the pandemic and plateaued at a higher level after the pandemic in Fall 2021. A similar increase in the number of quizzes completed by students was observed for 2000 level courses during the pandemic from Fall 2019 to Fall 2020 but declined dramatically to a pre-pandemic level in Fall 2021, while no significant changes were observed for 3000 and 4000 level during the study period.

Aside from quizzes, the *Number of Assignment Submissions* feature provided another perspective on students’ commitment to course materials. In 1000, 3000, and 4000 level courses, the number of assignments submitted by students increased during the pandemic and plateaued at a higher level after the pandemic in Fall 2021. In contrast, the number of assignments completed in 2000 level courses dramatically increased during the pandemic from Fall 2019 to Fall 2020, and then, declined to a pre-pandemic level in Fall 2021.

Regardless of various trends in *Total Time Spent in Content*, *Number of Logins To The System* steadily decreased during the study period for all chemistry courses. Across various levels of courses, 3000 and 4000 level courses had the highest average number of logins to the LMS system.

*Discussion Post Created* feature was used to investigate how students interact with each other. The usage of discussion tools on LMS was close to zero before the pandemic in Fall 2019. In all chemistry courses, *Discussion Post Created* usage slightly increased during the pandemic (Spring 2020 to Spring 2021) and dropped back to a pre-pandemic level in Fall 2021. The continuing underutilization of discussion tools on the LMS revealed that students were not actively engaged with peer students via LMSs.

Lastly, in order to examine how instructors used LMS pre- and post-pandemic, *Content Required* feature was plotted in Figure 2. Even though 2000 level courses displayed the highest average usage during the study period, the usage in both 1000 and 2000 level courses significantly increased during the pandemic (Spring 2020 to Spring 2021) and persisted after the pandemic (Fall 2021). However, 3000 and 4000 level courses returned to similar levels of usage after the pandemic in Fall 2021. This indicates that instructors in all chemistry courses adapted to the changes during the pandemic and utilized LMS more to support the teaching. The instructors in 1000 and 2000 level courses continued to utilize LMS at a higher level even after the pandemic while those of 3000 and 4000 level courses returned to their pre-pandemic level of usage in Fall 2021.

## Implications

A total of four features (*Quiz Completed*, *Number of Assignment Submissions*, *Total Time Spent in Content*, and *Number of Logins To The System*) were used to assess students’ engagement with course materials.^23−26^ Overall, students were more engaged with course materials in 1000 level courses, even after the pandemic. After resuming in-person instructions, the chemistry department kept a few online sections of 1000 level courses for students enrolled in the online Information Technology degree at GGC, which may lead to the higher-level engagement after the pandemic. Students in 2000 level courses were more active during the pandemic, but their activities soon returned to the pre-pandemic level in Fall 2021. Lastly, students’ commitment in 3000 and 4000 level courses did not change much during and after the pandemic.

The students’ interaction with peer students on the LMS (evaluated by *Discussion Post Created*) slightly increased during the pandemic and dropped to close to zero after in-person and hybrid courses resumed, which is consistent with other studies.^27−29^ Even during the pandemic, instructors may choose to use real-time tools, such as Breakout Groups during online class meetings for interactions among peer students. The in-person discussion took place in classrooms and laboratories may have completely replaced the discussion tools on the LMS after the pandemic.

The instructors in lower-level courses continued to use the LMS after the pandemic as extensively as during the pandemic, while those in upper-level courses returned to their pre-pandemic level of usage in Fall 2021. Many instructors, especially in 1000 and 2000 level courses, prepared prerecorded videos for students during the pandemic,^10^ and they may have chosen to continue to offer these resources after the pandemic. However, the instructors for 3000 and 4000 level courses in the chemistry department rotate off after two semesters of teaching the same course, which could be the factor that leads to the difference in the usage patterns. The instructors in more recent semesters may not have the need to prepare prerecorded videos, therefore, the usage of *Content Required* feature on the LMS fell down to pre-pandemic level.

COVID-19 pandemic has certainly promoted distance learning in higher education. During the pandemic, a huge amount of data was generated from various online teaching/learning tools. Our study takes advantage of these big data in a way that would benefit instructors, departments, and institutions.^30,31^ For individual instructors, we provide a new perspective to track and adjust the LMS usage in their course design throughout the semester. At the departmental level, new teaching/learning tools can be customized and adopted based on aggregated students’ engagement data from LMSs. At the institutional level, frequent reviews of LMS usage data can assist the administration to make data-driven decisions such as increasing the number of sections in a specific modality and expanding certain academic programs in response to the rapidly growing demands in online education. It is possible that LMS log data alone did not capture every tool that instructors used to facilitate teaching during and after the pandemic. Still, as the primary platforms to communicate with students, LMSs provide a macroscopic picture of how the pandemic has changed chemical education.

## Conclusions

This study analyzed the log data from LMS to investigate the evolution of LMS usage before, during, and after the pandemic. The first research question was “Do students engage with course materials and peer students on LMS differently before, during, and after the pandemic?” Students were not active in interacting with peer students on the LMS throughout the study period. In terms of engagement with the course materials, students all display unique patterns in different levels of courses. Students were more engaged with course materials in 1000 level courses during the pandemic, and that change carried over even after the pandemic. For 2000 level courses, students were more active during the pandemic but soon receded to pre-pandemic level in Fall 2021. For the 3000 and 4000 level courses, student engagement with course materials remained at almost the same level during and after the pandemic.

The second research question was “Do instructors utilize LMS differently after the pandemic?” Lasting change was observed for instructors in lower-level courses (1000 and 2000 level courses). They continued to use LMS extensively after the pandemic. In contrast, instructors in upper-level courses (3000 and 4000 level courses) rebounded to their pre-pandemic level of usage after resuming the face-to-face instructions.

The findings can be further utilized to customize the level-specific course design and facilitate student engagement through the optimal use of LMSs, eventually to ensure enhanced students’ learning experiences.

## Supporting Information Available

Detailed information about courses offered at various levels, LMS usage, description of LMS features, and academic performance (PDF, DOCX)
