# Supplementary material for: Differential Usage of Learning Management Systems in Chemistry Courses in the Time after COVID-19
Source: J Chem Educ. 2023 Apr 21;100(5):2033–8. doi: 10.1021/acs.jchemed.2c00850 (PMC10173450; doi:10.1021/acs.jchemed.2c00850)
Supplement: Supplementary file 2 — ed2c00850_si_002.docx [file ed2c00850_si_002.docx]

Supporting Information

**Differential Usage of Learning Management Systems in Chemistry Courses in the Time after COVID-19**

Ying Guo^a^*, Daniel Lee^b^

^a^ Department of Chemistry, School of Science and Technology, Georgia Gwinnett College, 1000 University Center Ln, Lawrenceville Georgia 30043 USA

^b^ STEM Academy, George Walton Comprehensive High School, 1590 Bill Murdock Rd, Marietta, Georgia 30062 USA

*E-mail: yguo1@ggc.edu

Page S2: Detailed list of courses offered at various levels, Table S1

Page S3: Detailed information about courses offered at various levels, Figure S1

Pages S4-6: Detailed information about LMS usage, Tables S2-4

Page S7: Description of LMS features

Page S8: Academic performance, Figure S2 & Table S5

Detailed list of courses Offered at various levels

Table S1. Detailed list of courses offered at various levels

| Level | Course Name |
| --- | --- |
| 1000 | CHEM1211K: Principles of Chemistry I with Laboratory  CHEM1212K: Principles of Chemistry II with Laboratory  CHEM1151K: Survey of Chemistry I with Laboratory  CHEM1152K: Survey of Chemistry II with Laboratory |
| 2000 | CHEM2211K: Organic Chemistry I with Laboratory  CHEM2212K: Organic Chemistry II with Laboratory |
| 3000 and 4000 | CHEM3000K: Analytical Chemistry with Laboratory  CHEM3100K: Inorganic Chemistry with Laboratory  CHEM3200: Environmental Chemistry  CHEM3300: Bioinorganic Chemistry  CHEM3500: Industrial Chemistry  CHEM4000: Chemistry Special Topics  CHEM4100K: Instrumental Chemistry with Laboratory  CHEM4201K: Physical Chemistry I with Laboratory  CHEM4202K: Physical Chemistry II with Laboratory  CHEM4550: Polymer Chemistry  CHEM4701: Integrated Lab I  CHEM4702: Integrated Lab II |

Detailed Information about courses Offered at various levels

In our department, teaching load for faculty is between two to three courses each semester. On average, there were 40 – 50 instructors teaching all levels of courses every semester during the study period. The decrease in enrollment was also reflected on the number of sections offered for each semester (Figure S1). During the study period, the number of sections offered for all chemistry courses constantly decreased from Fall 2019 to Spring 2021 with a slight increase in Fall 2021. Among all courses, 1000 level courses designed for all science majors (natural sciences, math, technology or health sciences), were affected the most. During the pandemic, the sections with low enrollment would be closed before the semester started and the resultant number of sections was lower. Since 3000 and 4000 level courses were required for chemistry majors to graduate, courses would still run despite low enrollment. Therefore, 3000 and 4000 level courses were not affected as much as 1000 or 2000 level courses, except for in Spring 2021 semester.

Figure S1. Number of courses offered at 1000 level (blue), 2000 level (red), and 3000 and 4000 level (green) from Fall 2019 to Fall 2021 semesters. Total number of sections offered for each semester was labeled at the top.

Detailed information about LMS usage

LMS usage data of all features were normalized using the min-max method. Average values of normalized usage data of all LMS features were summarized in Tables S2-4.

Table S2. Normalized average usage data for 1000 level courses.

| Year | Semester | Content Completed | Content Required | Checklist Completed | Quiz Completed | Total Quiz Attempts | Discussion Post Created | Discussion Post Replies | Discussion Post Read | Number Of Assignment Submissions | Total Time Spent In Content | Number Of Logins To The System |
| --- | --- | --- | --- | --- | --- | --- | --- | --- | --- | --- | --- | --- |
| 2019 | Fall | 0.0751 | 0.1668 | 0.0003 | 0.0831 | 0.0142 | 0.0001 | 0.0000 | 0.0000 | 0.0074 | 0.0289 | 0.0823 |
| 2020 | Spring | 0.0926 | 0.1978 | 0.0001 | 0.1346 | 0.0214 | 0.0071 | 0.0036 | 0.0007 | 0.0428 | 0.0389 | 0.0836 |
| 2020 | Fall | 0.1301 | 0.2571 | 0.0077 | 0.2060 | 0.0297 | 0.0159 | 0.0058 | 0.0017 | 0.1158 | 0.0544 | 0.0736 |
| 2021 | Spring | 0.1196 | 0.2504 | 0.0073 | 0.1786 | 0.0267 | 0.0178 | 0.0026 | 0.0011 | 0.0984 | 0.0481 | 0.0734 |
| 2021 | Fall | 0.1290 | 0.3003 | 0.0069 | 0.1626 | 0.0292 | 0.0073 | 0.0003 | 0.0002 | 0.0971 | 0.0454 | 0.0545 |

Table S3. Normalized average usage data for 2000 level courses.

| Year | Semester | Content Completed | Content Required | Checklist Completed | Quiz Completed | Total Quiz Attempts | Discussion Post Created | Discussion Post Replies | Discussion Post Read | Number Of Assignment Submissions | Total Time Spent In Content | Number Of Logins To The System |
| --- | --- | --- | --- | --- | --- | --- | --- | --- | --- | --- | --- | --- |
| 2019 | Fall | 0.1309 | 0.2009 | 0.0000 | 0.0429 | 0.0070 | 0.0000 | 0.0000 | 0.0000 | 0.0267 | 0.0489 | 0.1341 |
| 2020 | Spring | 0.1486 | 0.2824 | 0.0000 | 0.0550 | 0.0064 | 0.0004 | 0.0003 | 0.0001 | 0.1089 | 0.0571 | 0.1250 |
| 2020 | Fall | 0.2095 | 0.3556 | 0.0113 | 0.1409 | 0.0174 | 0.0073 | 0.0098 | 0.0068 | 0.1905 | 0.1219 | 0.1212 |
| 2021 | Spring | 0.1407 | 0.2802 | 0.0007 | 0.1567 | 0.0168 | 0.0064 | 0.0016 | 0.0003 | 0.1414 | 0.0746 | 0.1174 |
| 2021 | Fall | 0.1589 | 0.3323 | 0.0014 | 0.0273 | 0.0032 | 0.0018 | 0.0020 | 0.0007 | 0.0535 | 0.0664 | 0.1030 |

Table S4. Normalized average usage data for 3000 and 4000 level courses.

| Year | Semester | Content Completed | Content Required | Checklist Completed | Quiz Completed | Total Quiz Attempts | Discussion Post Created | Discussion Post Replies | Discussion Post Read | Number Of Assignment Submissions | Total Time Spent In Content | Number Of Logins To The System |
| --- | --- | --- | --- | --- | --- | --- | --- | --- | --- | --- | --- | --- |
| 2019 | Fall | 0.1066 | 0.1471 | 0.0000 | 0.0344 | 0.0039 | 0.0003 | 0.0000 | 0.0002 | 0.0583 | 0.0535 | 0.1578 |
| 2020 | Spring | 0.1644 | 0.1992 | 0.0000 | 0.0752 | 0.0102 | 0.0195 | 0.0048 | 0.0025 | 0.0569 | 0.0770 | 0.1625 |
| 2020 | Fall | 0.1295 | 0.1726 | 0.0000 | 0.0609 | 0.0066 | 0.0136 | 0.0045 | 0.0030 | 0.1321 | 0.0596 | 0.1637 |
| 2021 | Spring | 0.2218 | 0.2829 | 0.0000 | 0.0499 | 0.0063 | 0.0014 | 0.0003 | 0.0002 | 0.1475 | 0.0896 | 0.1562 |
| 2021 | Fall | 0.1020 | 0.1384 | 0.0000 | 0.0562 | 0.0079 | 0.0000 | 0.0000 | 0.0000 | 0.1166 | 0.0563 | 0.1453 |

DeScription of LMS features

*Quiz Completed:* Total number of quizzes completed by students on LMS for a specific course

*Number of Assignment Submissions:* Total number of assignments by students on LMS for a specific course

*Total Time Spent in Content:* Total amount of time (in minutes) students spend viewing “Content” posted by instructors on LMS for a specific course

*Number of Logins To The System:* Total number of times that students logged into the LMS system

*Discussion Post Created:* Total number of posts created by students on LMS for a specific course

*Content Required:* Total number of items posted by instructors as “Content” category and marked as required on LMS. Examples of “Content” items include but are not limited to links to external reading materials, embedded audio/video files, and reading materials directly posted by instructors.

academic performance

Figure S2. Final grades for 1000 level courses (blue), 2000 level courses (red), and 3000 & 4000 level chemistry courses (green) from Fall 2019 to Fall 2021 semesters.

Final grades of courses at different course levels were shown in Figure S2. Means and standard deviations of final grades for students enrolled in different levels of chemistry courses were summarized in Table S5.

Table S5. Average final grades of students in chemistry courses from Fall 2019 to Fall 2021.

| Year | Semester | 1000 Level Courses | 2000 Level Courses | 3000 and 4000 Level Courses |
| --- | --- | --- | --- | --- |
| 2019 | Fall | 76.1 ± 18.6 | 80.4 ± 16.9 | 85.0 ± 15.4 |
| 2020 | Spring | 80.5 ± 16.5 | 81.7 ± 17.8 | 85.8 ± 12.2 |
| 2020 | Fall | 71.8 ± 24.0 | 76.8 ± 19.4 | 83.5 ± 13.9 |
| 2021 | Spring | 71.3 ± 24.5 | 76.9 ± 21.6 | 83.3 ± 18.4 |
| 2021 | Fall | 73.8 ± 21.0 | 79.2 ± 18.7 | 80.7 ± 19.4 |

One-way ANOVA was used to determine if the average final grades were statistically different during and after the pandemic. At a significance level of 0.05, no statistical difference was detected during the study period for 2000 level courses (p = 0.082) and 3000 and 4000 level courses (p = 0.187). For 1000 level courses, average final grades were significantly different during and after the pandemic (p = 8.29 × 10^-36^). This indicates that the changes in modality of instructions during the pandemic did not adversely affect student learning for 2000, 3000 and 4000 level courses but did impact 1000 level courses.
